# Posterior fossa ependymoma: a comprehensive review of molecular classification, management guidelines, and clinical outcomes (Part I of ependymomas across compartments)

**DOI:** 10.1007/s11060-026-05808-9

**Published:** 2026-09-22

**Authors:** George W. Koutsouras, Francisco Rivera, Anthony M. Price, Derek S. Tsang, Adam Esbenshade, Vijay Ramaswamy, Peter B. Dirks, Michael C. Dewan

**Affiliations:** 1 Department of Neurological Surgery, Vanderbilt University Medical Center, Nashville, TN USA; 2 Division of Pediatric Neurosurgery, Monroe Carell Jr. Children’s Hospital at Vanderbilt University Medical Center, Nashville, TN USA; 3 Department of Neurosurgery, Barrow Neurological Institute, St. Joseph’s Hospital and Medical Center, Phoenix, AZ USA; 4 Radiation Medicine Program, Princess Margaret Cancer Centre, University Health Network, Toronto, ON Canada; 5 Department of Pediatrics, Division of Pediatric Hematology/Oncology, Vanderbilt University Medical Center, Nashville, TN USA; 6 Section of Neuro-Oncology, Division of Hematology/Oncology, The Hospital for Sick Children, Toronto, ON Canada; 7 Departments of Pediatrics and Medical Biophysics, University of Toronto, Toronto, ON Canada; 8 Division of Neurosurgery, The Hospital for Sick Children, Toronto, ON Canada; 9 Departments of Surgery and Molecular Genetics, University of Toronto, Toronto, ON Canada

**Keywords:** Pediatric ependymoma, Pediatric posterior fossa ependymoma, PFA, PFB

## Abstract

**Purpose:**

To review the clinical, molecular, and imaging landscape of posterior fossa (PF) ependymoma and outline current management principles guiding surgical and adjuvant treatment decisions.

**Methods:**

We performed a narrative review of the contemporary literature on molecular classification, imaging characteristics, and treatment outcomes of PF ependymoma, focusing on distinctions between posterior fossa group A (PFA) and group B (PFB) tumors.

**Results:**

PFA and PFB ependymomas are molecularly and clinically distinct. PFA tumors predominate in younger children, follow a more aggressive course, and show loss of H3K27me3, EZHIP overexpression, Moreover, in PFA tumors 1q gain and/or 6q loss defines a very high-risk group with increased recurrence and metastasis. PFB tumors arise more often in older patients, generally retain H3K27me3, harbor numerous arm-level chromosomal gains/losses, and carry more favorable outcomes. On MRI, PFA tumors more frequently show larger volume, hydrocephalus, lateral extension, skull base involvement, and lower apparent diffusion coefficient values. Gross total resection remains the most important prognostic factor and should be pursued whenever safe. Focal postoperative radiotherapy may be standard for localized disease, while chemotherapy has only a limited adjunctive role. Recurrence, especially among PFA tumors, is managed with early detection, repeat resection, and selective re-irradiation; 1q gain and 6q loss are enriched at relapse.

**Conclusions:**

PF ependymoma comprises two biologically and clinically distinct entities. Maximal safe resection and focal radiotherapy remain the backbone of treatment, but integrating molecular subgrouping, imaging biomarkers, and modern radiotherapeutic techniques into surgical and adjuvant planning offers the clearest path to improved outcomes, particularly for high-risk PFA tumors.

## Introduction

Ependymal tumors are rare primary central nervous system tumors, accounting for approximately approximately 5% pediatric CNS tumors; nearly 2/3rd of pediatric ependymomas arise in the posterior fossa [1–3]. Moreover, nearly 45% of affected children ultimately develop recurrent or refractory disease that remains difficult to cure [4]. Despite a relatively uniform histologic appearance, ependymomas are biologically heterogenous, with distinct epigenetic landscapes, developmental origins, and clinical outcomes [5]. Molecular profiling has shifted the World Health Organization (WHO) classification from a predominantly histologic framework toward integrated molecular entities defined entities, including Posterior Fossa ependymoma group A (PFA) and Posterior Fossa ependymoma group B (PFB) [6, 7]. 

Posterior fossa ependymomas (PFE) comprise PFA, PFB, and rarely subependymoma. The latter occurs almost exclusively in adults and is not relevant to the pediatric population. As part of a three-part series on ependymomas by anatomic compartment, this narrative review focuses on posterior fossa ependymoma, with emphasis on the PFA and PFB subgroups. We synthesize current evidence on biology, imaging, management, recurrence, and molecular stratification in risk-adapted care.

## Molecular landscape

**Fig. 1:**
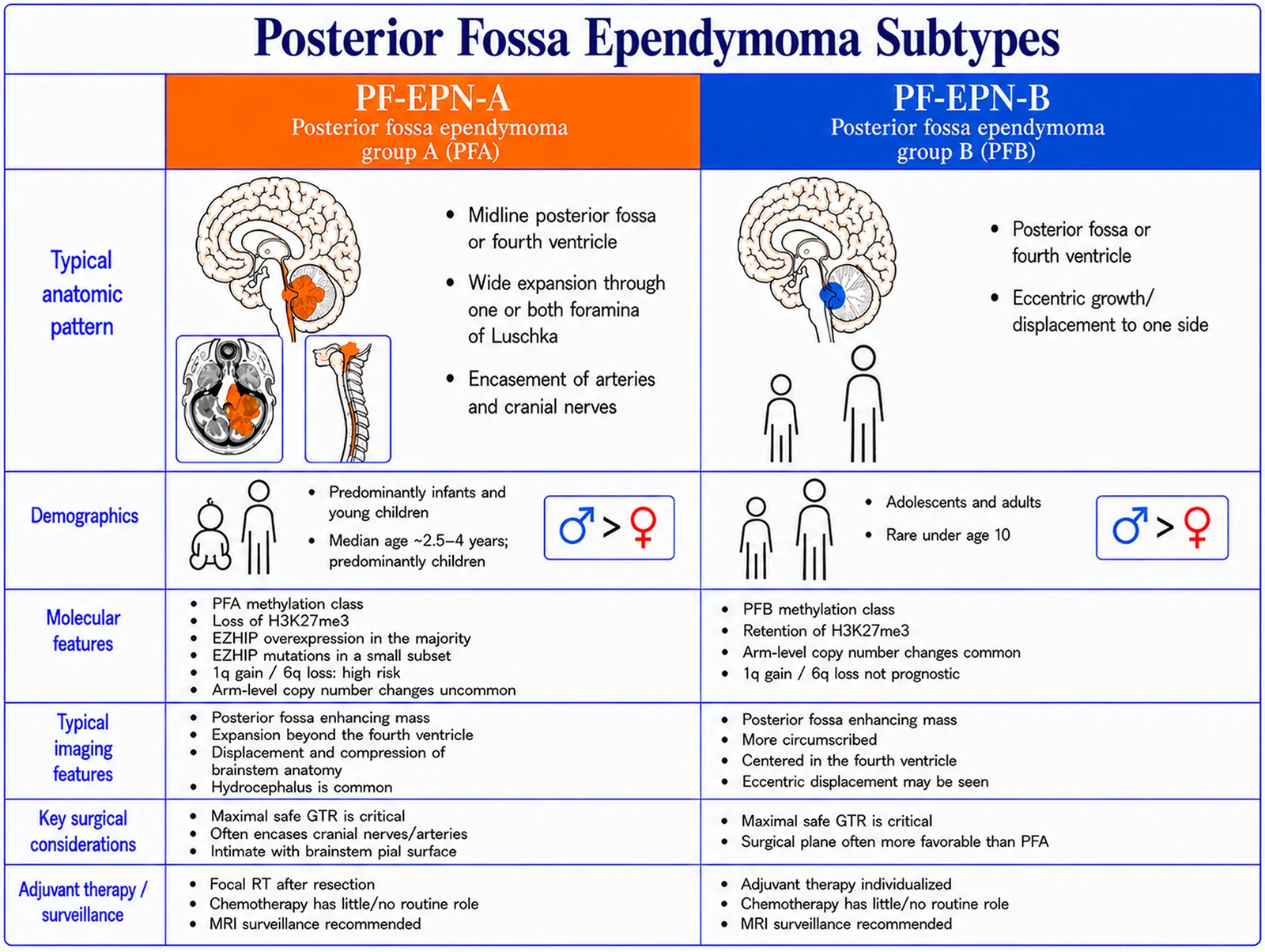
Posterior fossa ependymoma subtypes. Abbreviations: CNS, central nervous system; WHO, World Health Organization; CNS WHO, World Health Organization Classification of Tumors of the Central Nervous System; PF-EPN-A, PFA, posterior fossa ependymoma group A; PF-EPN-B, PFB, posterior fossa ependymoma group B; H3K27me3, histone H3 lysine 27 trimethylation; MRI, magnetic resonance imaging; RT, radiotherapy

Large-scale genomic studies have advanced understanding of PFE biology. Transcriptomic analyses showed that these tumors represent biologically distinct diseases despite similar histopathology [5]. Genome-wide DNA methylation profiling refined this concept and established molecular classification across ependymal tumors, forming the basis for the current WHO framework [6, 8]. WHO classifications have evolved from a 2007 histology-based system to a molecularly integrated framework [5, 6, 9]. Consequently, PFE are now recognized as two principal molecular subgroups, PFA and PFB, with distinct molecular and clinical characteristics that provide greater prognostic insight than histologic grading alone (Fig. 1) [6, 10]. 

Epigenetic dysregulation largely drives the biological distinction between these subgroups. PFA tumors demonstrate global reduction of repressive histone 3 lysine 27 trimethylation (H3K27me3) due to inhibition of the polycomb repressive complex 2 (PRC2), commonly mediated by overexpression of enhancer of zeste homolog inhibitory protein (EZHIP) [11, 12]. This epigenetic alteration has been shown to correlate with worse clinical outcomes and serves as an important diagnostic marker [13]. Among PFA tumors, 1q gain and/or 6q loss define a very high-risk subset associated with recurrence, metastatic progression, and poor survival despite gross total resection (GTR) and upfront radiotherapy [14–16]. 

PFB ependymomas demonstrate distinct molecular features. In contrast to PFA, PFB ependymomas retain global H3K27me3, reflecting preserved PRC2-mediated regulation and have much higher degrees of somatic aneupleudy [13, 17]. Throughout this review, we will highlight the impact of molecular classification on disease management, imaging phenotype, resectability, treatment response, and outcomes.

## Clinical presentation

Posterior fossa ependymomas in children commonly present with increased intracranial pressure from obstructive hydrocephalus [18]. Typical presenting symptoms include headache, nausea, vomiting, lethargy, and gait disturbance [19]. Moreover, they are often accompanied by truncal ataxia reflecting cerebellar involvement [19]. Symptom onset may be insidious; a long history of nausea or vomiting without headache often leads to delayed diagnosis. Lower cranial nerve deficits may also be present.

**Fig. 2:**
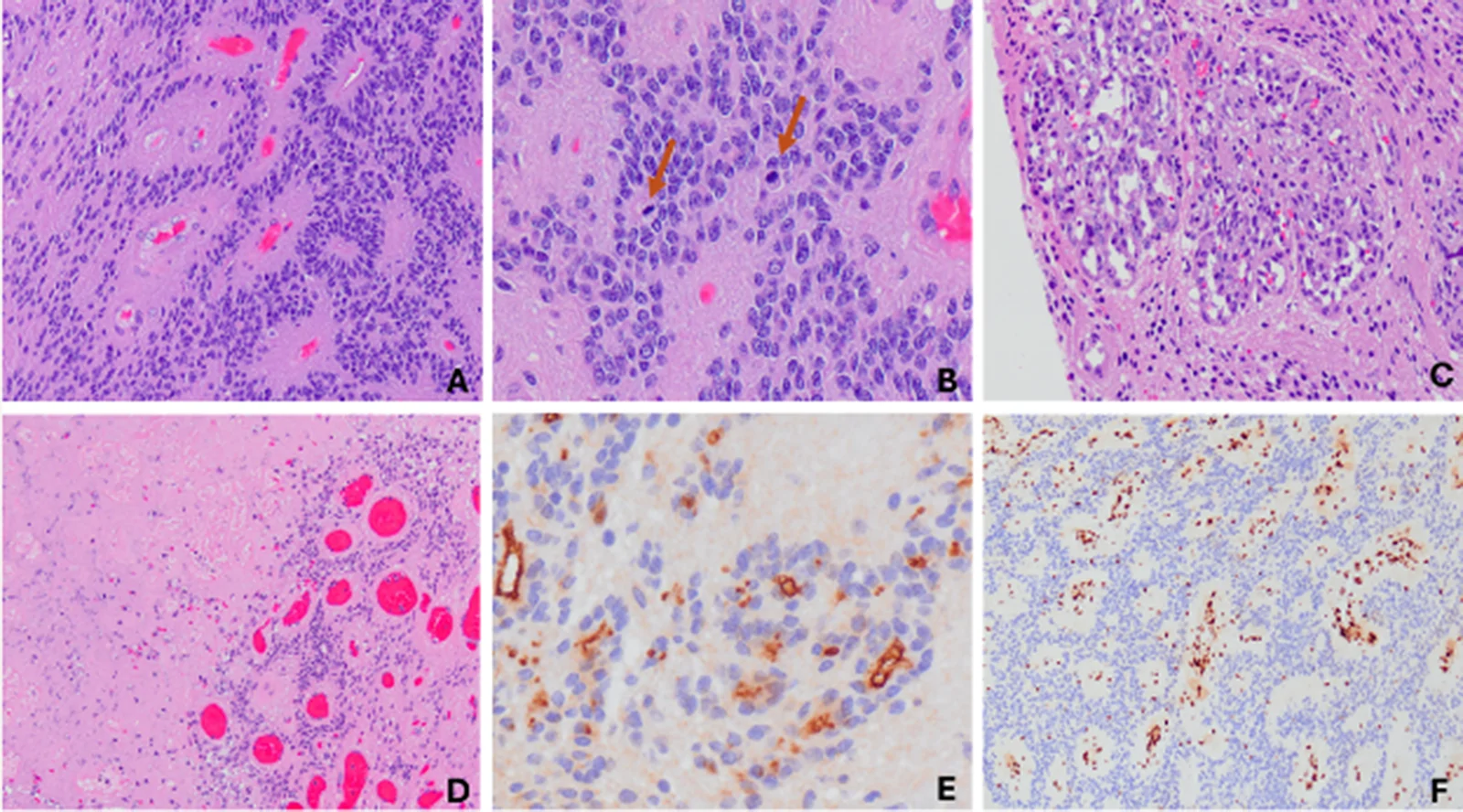
Posterior Fossa Group A Ependymoma: Histologic and Immunohistochemical Features. (**A**; 200×) Relatively monomorphic cells are present with round to ovoid nuclei, finely dispersed chromatin, and inconspicuous nucleoli, arranged in prominent perivascular pseudorosettes. (**B**; 400×) Brisk mitotic activity is present, with up to 10 mitotic figures (orange arrows) identified per 2 mm², reflecting high proliferative activity. (**C**; 200×) Microvascular proliferation is present, characterized by multilayering of endothelial cells and glomeruloid vascular structures. (**D**; 100×) Focal necrosis is identified, with associated apoptotic debris. (**E**; 400×) Immunohistochemistry for EMA highlights characteristic dot-like paranuclear and luminal staining. (**F**; 100×) Tumor cells show loss of nuclear expression of H3 p.K28me3 (K27me3), supporting classification as posterior fossa group A ependymoma

**Fig. 3:**
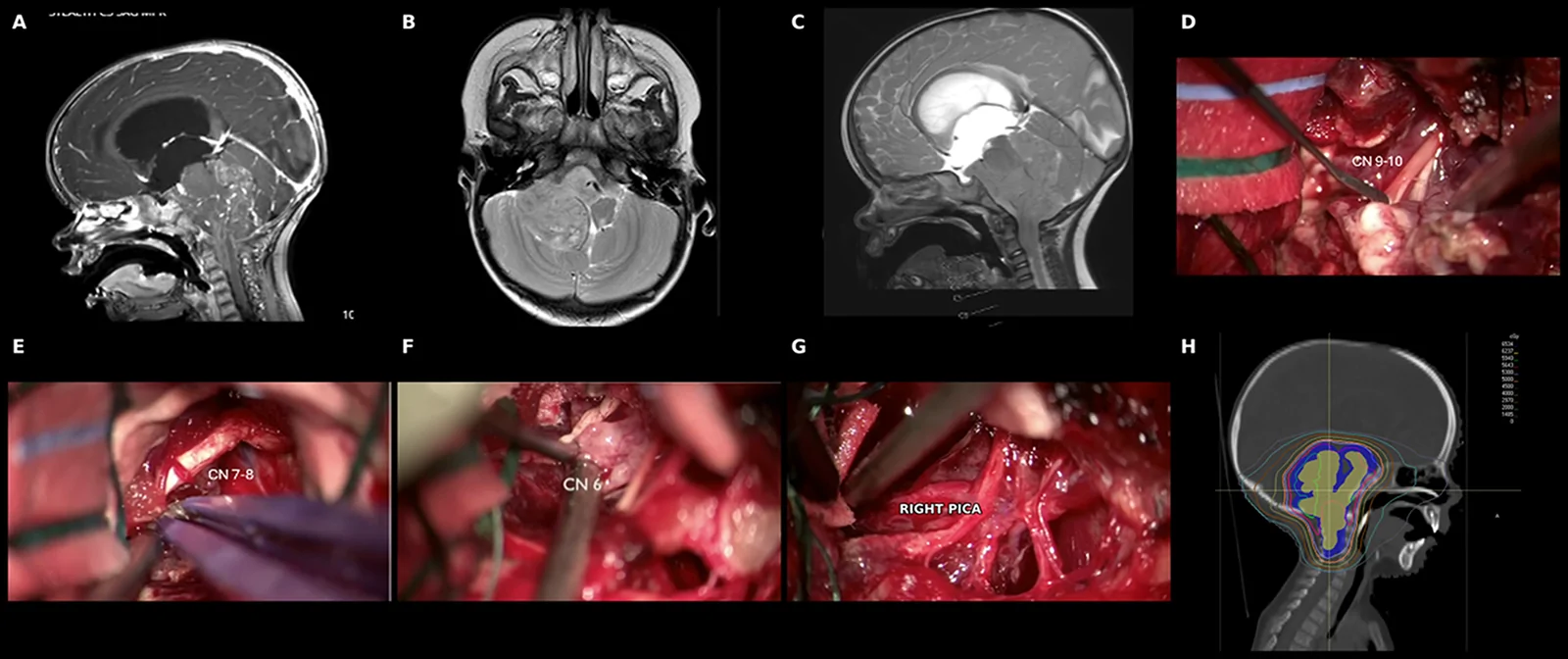
Multimodal imaging, intraoperative neurovascular anatomy, and radiotherapy planning of a posterior fossa group A (PFA) ependymoma in an 11-month-old girl. Clinical vignette. An 11-month-old female presented with evidence of papilledema and underwent non-contrast CT head imaging that demonstrated a large fourth ventricular lesion with extension into the posterior fossa cisterns and ventral to the spinal cord. MRI demonstrated a mixed, heterogeneously enhancing mass. The patient was clinically stable. The operative plan was to position the patient prone, with the face well-padded on a horseshoe head rest. A Frazier’s point external ventricular drain was inserted with neuronavigation, and a suboccipital craniotomy (right more than left), with C1 and partial C2 laminectomy, was performed. Intraoperatively, the lesion was seen expanding the tela choroidea through the foramen of Magendie and out the right foramen of Luschka. The lesion encased cranial nerves IV through XII and the vertebrobasilar system. A gross total resection was performed. Pathology was consistent with posterior fossa (PF) group A ependymoma, demonstrating monomorphous ovoid nuclei arranged in perivascular pseudorosettes and true rosettes, with necrosis seen. Next-generation sequencing demonstrated a mutation in the KMT2C gene; no 1q gain or 6q loss was observed, and H3K27me3 was lost in tumor cells. The patient had a left cranial nerve VI and right cranial nerve VII palsy, which has slowly improved at 6 months. However, at 6 months, MRI demonstrated recurrent lesions in the lumbar spine, with pathology demonstrating recurrent PFA ependymoma with loss of H3K27me3 but without evidence of a KMT2C genetic mutation. Panels. (**A**) Sagittal post-contrast T1-weighted MRI showing a heterogeneously enhancing mass filling the fourth ventricle with extension into the posterior fossa cisterns and ventral to the cervicomedullary junction. (**B**) Axial and (**C**) sagittal T2-weighted MRI demonstrating the mixed-signal lesion and associated ventricular dilation. (**D–G**) Intraoperative microscopic views showing the tumor densely adherent to (**D**) cranial nerves IX–X, (**E**) the VII–VIII complex within the cerebellopontine angle and porus acousticus, (**F**) cranial nerve VI, and (**G**) the right posterior inferior cerebellar artery (PICA); meticulous microdissection with fine-tip forceps, suction, and microdissectors enabled a gross total resection. (**H**) Postoperative sagittal photon radiotherapy plan: the phase 1 target was treated to 54 Gy (blue color wash) with a sequential conedown boost to 59.4 Gy (yellow colorwash), while the spinal cord dose was constrained below 54 Gy

Presentation varies by molecular subgroup. PFA tumors typically arise in younger children (median age 5 years) and are associated with larger tumor burden and more pronounced hydrocephalus [20]. Figures 2 and 3 provide a histopathologic review of PFA subtype and a clinical vignette. In contrast, PFB tumors occur in adolescents and adults and present less aggressively [20]. Although symptom patterns overlap, these differences parallel the distinct biological behavior of each subgroup.

## Neurological imaging

Magnetic resonance imaging (MRI) is the principal imaging modality for the evaluation of PFE and remains essential for diagnosis, operative planning, and surveillance [21]. Tumors typically arise from the fourth ventricle, with heterogeneous signal, cystic change, calcification, variable enhancement, and characteristic extension through the foramina of Luschka and Magendie [22, 23]. 

Imaging phenotype may also reflect molecular subgroup [24]. PFA tumors are generally larger, more often associated with hydrocephalus and lateral or skull-base extension, and demonstrate lower ADC values, whereas PFB tumors are typically more homogeneous, circumscribed and cystic [21, 24]. Craniospinal MRI should be obtained at diagnosis, during surveillance, and at recurrence; thin-cut T2-weighted spinal imaging is important because metastatic deposits may not enhance, while T2-FIESTA sequences may aid operative planning.

## Surgical management

**Fig. 4:**
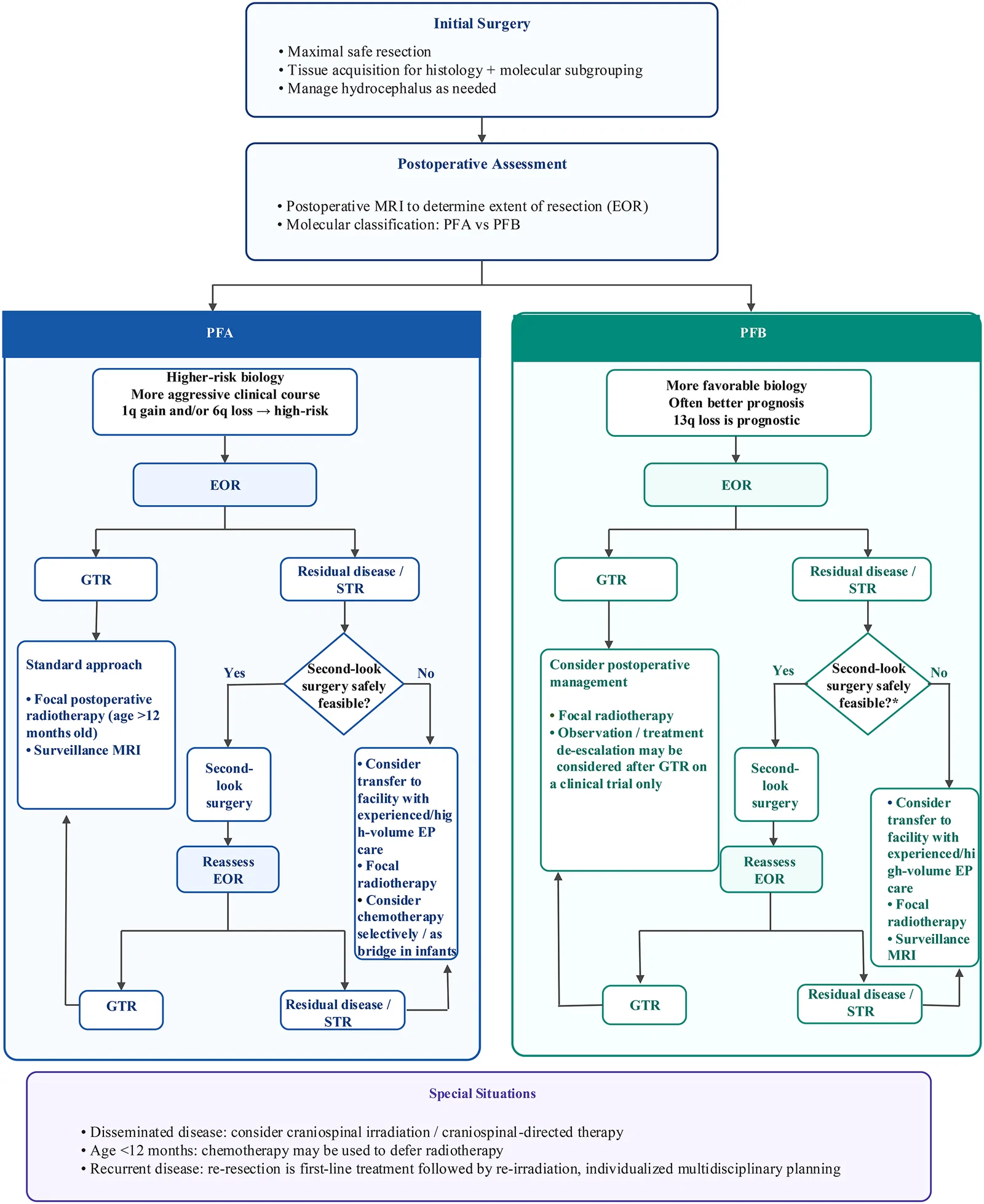
Proposed risk-adapted clinical management algorithm for pediatric posterior fossa ependymoma. following maximal safe resection and postoperative molecular subgrouping, management is stratified by molecular subtype (PFA vs. PFB) and extent of resection (EOR). For PFA tumors, GTR is followed by focal postoperative radiotherapy in children older than 12 months and surveillance MRI. Residual disease prompts consideration of second-look surgery when safely feasible; when further resection is not possible, focal radiotherapy is initiated with selective chemotherapy considered as a bridge in infants. For PFB tumors, GTR is followed by focal radiotherapy, with observation or treatment de-escalation considered only within the context of a clinical trial. Residual PFB disease is managed with second-look surgery when feasible, followed by focal radiotherapy and surveillance. Special situations, including disseminated disease, age younger than 12 months, and recurrent disease, often require individualized multidisciplinary planning with re-resection and re-irradiation when feasible. Maximal safe resection and focal postoperative radiotherapy are standard, whereas radiotherapy de-escalation in PFB and molecularly directed therapy remain investigational. This figure represents a conceptual management framework; all treatment decisions should be individualized in a multidisciplinary setting. Abbreviations: PFA, posterior fossa group A; PFB, posterior fossa group B; EOR, extent of resection; GTR, gross total resection; STR, subtotal resection; MRI, magnetic resonance imaging; RT, radiotherapy. *In high-risk PFA (1q gain / 6q loss) with STR, weigh re-resection morbidity against quality of life

Surgery is the most important component of PFE management, with extent of resection the key modifiable determinant of disease control (Fig. 4) [25]. Operative goals include GTR, tissue diagnosis, molecular characterization, and treatment of hydrocephalus when present. For isolated lesions at diagnosis, operative success is defined by complete resection [26]. Tissue acquisition is also essential for subgroup classification as prognostic effect of surgery is increasingly understood within the context of molecular subgroupings and high risk genomic alterations [27, 28]. For example, adverse PFA biology, particularly 1q gain and/or 6q loss, may attenuate the survival benefit associated with complete resection. The Children’s Oncology Group (COG) ACNS0121 study compared GTR followed by immediate conformal radiotherapy with STR followed by adjuvant therapy. The study demonstrated superior outcomes for the GTR and radiotherapy group with 5-year event-free survival of 68.5% vs. 37.2% and overall survival of 86.2% vs. 70.2% [29]. In a posterior fossa–specific study by Silva da Costa et al., GTR was associated with improved outcomes [30]. In their study, the median overall survival was 136 months after GTR compared with 38 months after STR, and the progression-free survival was similarly prolonged (40 vs. 7 months, respectively) [30]. However, ACNS0831 and other studies showed that a meaningful subset of patients had residual tumor, supporting consideration of second-look surgery when safely feasible [31, 32]. These associations should nevertheless be interpreted cautiously, because most surgical series are retrospective and extent of resection may partly reflect tumor anatomy, biological phenotype, institutional experience, and patient selection rather than an isolated treatment effect. Taken all together, we propose a conceptual framework for the clinical management of PF ependymoma in Fig. 4.

Residual disease should prompt early second-look surgery or referral to an experienced center when further safe resection is feasible [33, 34]. Complex ventral extension around the brainstem or multi-compartmental extension may occassionally require a staged operation. However, potential gains in local surgical control, whether in relation to second-look surgery or to a staged surgical approach, must be balanced against morbidity associated with delays in adjuvant therapy.

Surgical morbidity remains substantial because these tumors are closely related to the fourth ventricular floor, cranial nerve nuclei and fascicles, and posterior circulation vasculature [35]. In a recent molecularly defined pediatric cohort, cranial neuropathies occurred in 60% of patients after primary resection, although 56% resolved within 1 year [35]. Common deficits include cranial nerve palsies, dysphagia, limb weakness, and truncal ataxia, particularly affecting gait and swallowing [36]. Notably, neurological deficits may peak in the immediate postoperative period and generally improve over time [36]. These observations are relevant in PFA ependymomas, in which local control and survival depend strongly on GTR [26]. Accordingly, maximal safe resection should remain the primary surgical objective, and requires substantial surgical experience and commitment. Although neurovascular preservation is essential, residual tumor should not be left behind when a safe resection plane exists.

### Intraoperative surgical adjuncts and considerations

Most patients are positioned prone; head fixation should be individualized by patient age and calvarial thickness, as pin fixation in children younger than 2 years risks calvarial injury and poor stability. A horseshoe headrest with appropriate padding is suitable for midline approaches in young children, particularly those with a thin calvarium from progressive hydrocephalus [37]. 

Intraoperative neuromonitoring, particularly with spontaneous EMG recordings of cranial nerves, may help maximize resection while prioritizing safety. Neuronavigation with stereotaxy, ultrasound, and intraoperative MRI has proven useful in practice and observed in the literature [38, 39]. Furthermore, external ventricular drain placement should be considered because hydrocephalus occurs in up to 80% of children with a posterior fossa mass [40]. Ventricular access should be individualized by hydrocephalus, ventricular size, positioning, and surgical approach [41]. 

## Radiation therapy

Postoperative focal radiotherapy is central to local disease control and is recommended for most children with localized PFE after maximal safe resection [29, 42, 43]. Contemporary conformal radiotherapy achieves durable control, including in appropriately selected young children, supporting treatment in patients older than 12 months rather than routine deferral of radiation [44]. In the ACNS0121 study, the 5-year event-free survival for patients receiving immediate postoperative conformal radiotherapy after near-total or GTR was 68.5% [29]. Furthermore, outcomes in children younger than 3 years were comparable to older children [29]. These data support focal postoperative radiotherapy in children older than 12 months,

Typical doses are 59.4 Gy, with reduction to 54 Gy in selected very young patients after GTR [29, 31]. 

Modern conformal techniques, including proton therapy, reduce normal tissue exposure while maintaining local control. Contemporary COG protocols reduced clinical target volume margins from 10 mm in ACNS0121 to 5 mm in ACNS0831, limiting exposure to normal brain and cerebellum [29, 31]. Smaller CTVs reduce exposure to healthy brain and cerebellum, potentially reducing toxicity [45]. An example focal photon radiation plan is provided in Fig. 3.

Craniospinal irradiation is generally reserved for metastatic disease and/or at recurrence [27, 46]. Despite improvements in delivery, failure remains predominantly local. In the St. Jude series, local recurrence was more common than distant recurrence [44]. In SIOP Ependymoma I, focal radiotherapy (up to 59.4 Gy) followed surgery, often within a staged approach including second-look resection or chemotherapy, underscoring local control [46]. Thus, radiation efficacy depends on dose, technique, target definition, and extent of resection.

Proton therapy provides dosimetric advantages with disease control comparable to photon-based treatment in retrospective and multi-institutional series, and is regarded as a suitable adjuvant treatment after maximal safe resection of pediatric ependymoma [43, 47, 48]. There is no high quality data directly comparing toxicity among photon- and proton-treated children with ependymoma, however. In the absence of definitive data, photon radiotherapy remains an acceptable alternative when proton therapy is not readily available [49, 50]. Although the optimal photon RT dose for pediatric ependymoma is 59.4 Gy, there remains practice variation in proton radiation prescription doses, which range from 54 GyE to 59.4 GyE due to the potential risk of brainstem toxicity after proton therapy [51]; more data are needed to clarify optimal practice in future studies [52–54]. 

Molecular subgroups refine interpretation of radiation outcomes. PFA tumors have poorer survival than PFB tumors despite focal radiotherapy, suggesting intrinsic biology is a major outcome determinant [27]. In the SIOP Ependymoma I trial, adverse molecular markers—including 1q gain, hTERT expression, and H3K27me3 loss—were associated with inferior survival [46]. In ACNS0121, posterior fossa molecular subgrouping (PFA vs. PFB) did not independently stratify event-free survival among patients treated with conformal radiotherapy, whereas 1q gain, tumor grade and other clinical variables remained significant determinants of outcome [29]. For PF-EPN-A tumors in patients older than 12 months, maximal safe resection followed by focal radiotherapy is standard, ideally at specialized centers [55]. Favorable outcomes in gross totally resected PFB tumors support careful investigation of treatment de-escalation, including observation instead of upfront radiotherapy [55]. A multicentre cohort of PFB ependymoma showed that even in PFB ependymoma upfront radiotherapy significantly improves progression-free survival [56]. However, observation for PFB ependymoma should be approached with caution; PFB tumors still seem to derive a strong progression-free survival benefit with radiation compared to omission of radiation [56]. Thus, omission of RT in patients with PFB should only be carried out in a well-designed, prospective clinical trial. Together, current evidence supports a risk-adapted framework in which radiotherapy remains foundational but may be informed by molecular stratification in the future. Radiotherapy appears to improve local control across molecular subgroups; however, it does not completely overcome the high-risk biology of PFA tumors. Additionally, the patient age is closely correlated with molecular subtype and treatment strategy, and as PFA tumors predominant in younger children, the very young have historically been more likely to receive radiation deferral strategies. This, makes it difficult to distinguish from tumor biology and treatment exposure.

## Chemotherapy

Chemotherapy has little to no definitive role in PFE, including in infants, and should not be considered a substitute for maximal safe resection and focal radiotherapy. Early multiagent regimens delayed radiotherapy or treated residual disease, but none has shown clear benefit [55]. In ACNS0121, patients with subtotal resection (STR) received vincristine-, carboplatin-, cyclophosphamide-, and etoposide-based chemotherapy before reassessment and potential second surgery [29]. However, outcomes in this group remained inferior to those of patients treated with near-total or gross-total resection followed by immediate conformal radiotherapy [29]. This approach reflects the use of chemotherapy to facilitate local control rather than as definitive treatment. More recently, systemic targeted therapy has also shown limited efficacy. In ACNS1021 trial, sunitinib demonstrated no sustained objective responses, with predominantly mild-to-moderate adverse events [57]. 

Adjuvant chemotherapy was evaluated in the large randomized COG ACNS0831 trial, which compared postoperative radiation alone with radiation followed by four cycles of vincristine, cyclophosphamide, etoposide, and cisplatin [31]. The addition of chemotherapy did not improve outcomes across the overall cohort [31]. However, interpretation of COG chemotherapy studies is limited by the pooling of supratentorial and infratentorial tumors despite their distinct biology, and in older studies, by the absence of contemporary molecular classification, making posterior fossa specific treatment effects hard to isolate. As an emerging approach, Griesinger et al. identified chromosome 1q-gain PFA as a highest-risk subgroup and demonstrated preclinical sensitivity to 5-fluorouracil and all-trans retinoic acid, supporting future investigation of molecularly directed therapy [58]. Overall, focal radiation remains the preferred adjuvant therapy for most posterior fossa ependymomas in children older than 12 months. Chemotherapy is generally reserved for selected scenarios, particularly very young children in whom radiotherapy is being postponed.

Baby brain protocols (BabyPOG, UKCCSG CNS 9204, SFOP, HIT) achieved 5-year progression-free survival of up to 42% in children younger than 3 years, but are difficult to interpret given pre-molecular era design, absence of location stratification, and limited cytoreduction [55]. A large retrospective study of > 800 PFE showed that chemotherapy alone resulted in rapid progression and dismal survival. Accordingly, these baby brain protocols should be interpreted with caution specifically in PFA ependymoma [59]. In contrast, early conformal radiotherapy in similar populations resulted in substantially improved outcomes, with 7-year progression-free survival approaching 77% [55]. These data led to a shift away from radiation-deferral strategies in children older than 12 months.

## Recurrent disease

Recurrence remains a major challenge, occurring in up to 48% of cases with mortality approaching 50% despite multimodal therapy [28, 60–64]. Mean survival after first recurrence may be approximately 31 months [28]. However, reported dissemination rates range from 9% to 20%, with both supratentorial and spinal involvement observed at recurrence [61]. Recurrence may occur up to 10 years after completion of treatment, further emphasizing the need for prolonged follow-up [62]. 

Molecular subgroups are a key determinant of recurrence behavior and outcome. PFA tumors account for most recurrent cases and are associated with significantly worse post-progression survival compared to their PFB counterparts [28, 63]. In molecularly characterized cohorts, more than 80% of recurrent infratentorial ependymomas are PFA, with shorter survival following recurrence (24.7 months compared to 48 months) [28]. Tumor biology also evolves at recurrence, with increased frequency of high-risk genomic alterations. Chromosome 1q gain has been associated with reduced post-recurrence survival, with shorter latency observed in affected patients (14.7 vs. 25.3 months) [28]. Furthermore, loss of 6q has been shown to be enriched at recurrence in PFA ependymoma and may identify a subgroup at increased risk for subsequent relapse [63]. On the other hand, 13q loss may play a prognostic role in PFB tumors [56]. These patterns support surveillance strategies that include both intracranial and spinal imaging. Surveillance is not standardized, but most approaches incorporate serial MRI of the brain and spine over extended follow-up intervals given the risk of both local and disseminated recurrence [28, 61]. Furthermore, GTR at initial presentation may be associated with improved post-recurrence survival compared with STR [28]. However, in the same study, no difference in mortality was observed among patients with PFA tumors based on extent of resection at initial presentation [28]. 

Patients with PF tumors had better survival with GTR at presentation [59]. In this context, the primary goal of surveillance is early detection of resectable disease, as resectability at recurrence remains a key determinant of outcome. Surveillance intensity is tailored to clinical and molecular risk factors, including extent of resection and subgroup, to enable early identification of treatable disease and guide decisions regarding surgical salvage and craniospinal evaluation [28]. 

Treatment at recurrence is guided by resectability, disease distribution, and prior therapy. When recurrence is localized and safely accessible, repeat resection may be prioritized, as surgery at recurrence, including multiply recurrent disease, has been retrospectively associated with improved survival [28, 64]. After maximal safe re-resection, re-irradiation may be considered based on prior radiation exposure, time from initial treatment, patient age, and cumulative toxicity risk [65]. Craniospinal irradiation may be considered in disseminated recurrence, reflecting patterns of disease spread [66]. Surgery may be considered for isolated metastatic relapse, but craniospinal irradiation may still be required even after reoperation [67]. Mak et al. reported that craniospinal re-irradiation after local recurrence has better outcomes than focal re-irradiation in their cohort [64]. However, this cohort included ependymomas from multiple anatomical locations; the potential benefit from CSI re-irradiation for locally recurrent ependymoma seemed to be limited to posterior fossa primary tumors. Chemotherapy has demonstrated limited efficacy in this setting and is generally reserved for radiation-sparing purposes [28, 60]. Overall, treatment strategies emphasize maximal cytoreduction with operative re-resection, and repeat radiotherapy when feasible.

## Conclusions and future directions

Contemporary evidence supports an integrated model of posterior fossa ependymoma prognosis rather than reliance on any single clinical variable. Molecular subgroups and high risk copy number alterations, such as 1q and/or 6q loss, define biological risk, while gross total resection and focal radiotherapy remain the principal modifiable determinants of local control and survival. The relative contribution of age is less clear because of its close association with PFA biology and differences in treatment intensity. Importantly, many of these conclusions derive from retrospective, heterogenous and pre-molecular era cohorts, while prospective studies combine biologically distinct supratentorial and infratentorial tumors.

Future progress in pediatric PFE will require translating molecular classification into practical, risk-adapted treatment strategies. Priorities include defining the prognostic impact of high risk copy number alterations, optimizing local and diffuse control in PFA, evaluating treatment deescalation in PFB and developing molecularly-directed therapies. Advances in methylation profiling, liquid biopsy, imaging biomarkers, proton therapy, and multicenter prospective studies may further individualize treatment and surveillance while reducing long term toxicity.

## Data Availability

No datasets were generated or analysed during the current study.
